# Trichoepithelioma Arising in an Ovarian Mature Cystic Teratoma

**DOI:** 10.1155/2016/6829194

**Published:** 2016-12-29

**Authors:** Takashi Suzuki, Satoru Nakayama, Haruka Muto, Shinichi Shimizu, Yoshiro Otsuki, Hiroshi Adachi, Takeshi Murakoshi

**Affiliations:** ^1^Department of Obstetrics and Gynecology, Seirei Hamamatsu General Hospital, Shizuoka, Japan; ^2^Department of Clinical Laboratory, Ashikaga Red Cross Hospital, Ashikaga, Japan; ^3^Department of Pathology, Seirei Hamamatsu General Hospital, Shizuoka, Japan

## Abstract

Here, we report an extremely rare case of trichoepithelioma (TE)—a benign epithelial tumor originating from the outer root sheath of a hair follicle—arising in an ovarian mature cystic teratoma (MCT) with fluorodeoxyglucose-positron emission tomography (FDG-PET) findings. A 48-year-old Japanese woman presented to our hospital for her annual follow-up of adenomyosis. Ultrasonography and magnetic resonance imaging revealed a left ovarian tumor with irregular-shaped septum, which was suspicious of malignancy. However, tumor marker levels were within normal range. On FDG-PET, the maximum standardized uptake value (SUVmax) of the tumor was 2.9. Laparotomy with left salpingooophorectomy was performed. Pathologic examination revealed the probability of TE, rather than basal cell carcinoma (BCC), arising in an ovarian MCT. After five years of follow-up, the patient had no sign of recurrence. The FDG-PET SUVmax was low in TE, as with other benign tumor. However, future investigation is needed to evaluate the findings of FDG-PET imaging in TE cases.

## 1. Introduction

Trichoepithelioma (TE) is a benign epithelial tumor originating from the outer root sheath of a hair follicle, which usually develops on the skin of the head and neck. It can sometimes be confused with basal cell carcinoma (BCC)—an epithelial tumor arising from progenitor cells of the interfollicular epidermis and upper infundibulum [1]—even with immunohistochemical examination including CD10, CD34, Bcl-2, cytokeratin 15 and 20, D2-40, and androgen receptor [2–7]. Differentiation of the two is important because their treatment is significantly different.

Mature cystic teratoma (MCT) is the most common germ cell tumor, constituting 15% to 25% of all ovarian tumors. Approximately 1.5% of MCTs contain a malignant tumor, such as squamous cell carcinoma (75%), adenocarcinoma (7%), or BCC (<1%) [8], which is referred to as malignant transformation. There are also reports of MCTs containing a benign tumor, which is usually mucinous cystadenoma [9]. However, there are no reports of TE arising in an ovarian MCT.

There are numerous reports regarding the fluorodeoxyglucose-positron emission tomography (FDG-PET) findings of BCC [10–14], but there are no reports regarding the FDG-PET findings of TE, especially TE arising in an ovarian MCT. Here, we present an extremely rare case of TE arising in an ovarian MCT, which was confirmed by pathologic findings with FDG-PET findings.

## 2. Case Presentation

**Figure 1:**
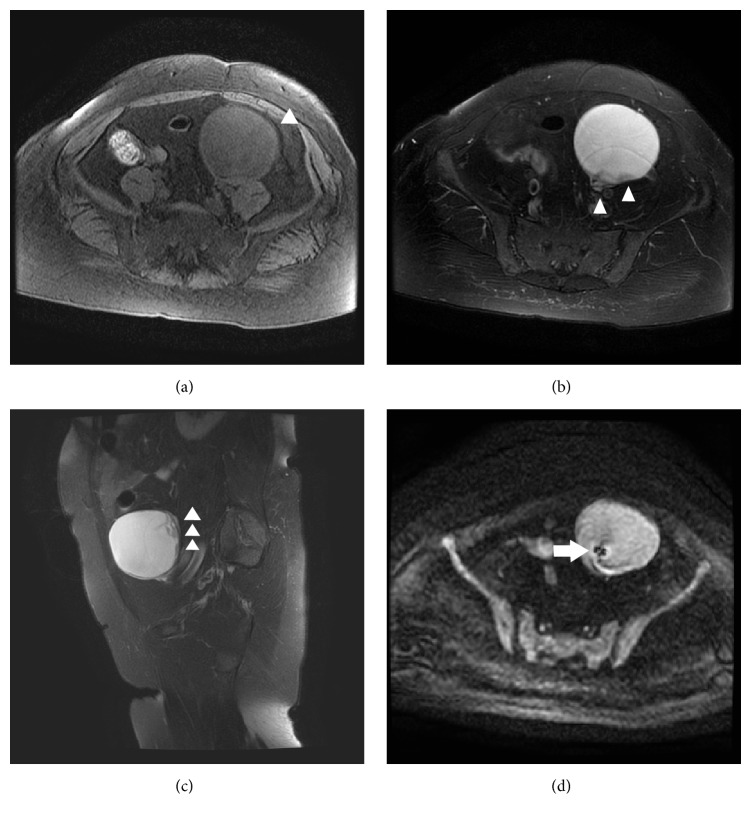
Magnetic resonance imaging findings. (a) T1-weighted transverse image, revealing an ovarian mature cystic teratoma (arrowhead). T2-weighted transverse (b) and sagittal (c) images, demonstrating heterogeneous signal intensity at the posterior wall (arrowheads). (d) Diffusion-weighted transverse image, demonstrating low signal intensity at the septum (arrow).

**Figure 2:**
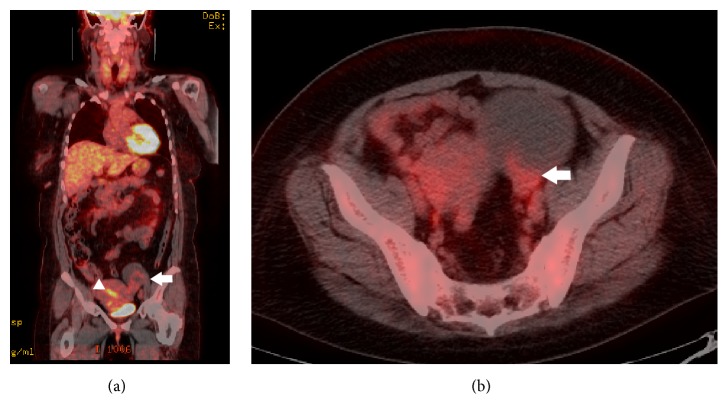
Fluorodeoxyglucose-positron emission tomography findings. Fused coronal (a) and transverse (b) images, demonstrating a maximum standardized uptake value of 2.9 in the ovarian mature cystic teratoma (arrow) (white arrowhead = endometrium).

A 48-year-old Japanese woman (gravida 2, para 2) presented to our hospital for her annual follow-up of adenomyosis. She had a history of bronchial asthma and schwannoma derived from the 9th, 10th, and 11th cranial nerves. During the previous year, there was no ovarian cyst, but recent ultrasonography showed a left ovarian cystic tumor with a solid portion, which was suspicious of malignancy. Levels of carcinoembryonic antigen, cancer antigen (CA) 19-9, CA125, CA72-4, and Sialyl Lewis X were 1.1 ng/mL, 30.9 U/mL, 12.7 U/mL, <3.0 U/mL, and 28 U/mL, respectively. Magnetic resonance imaging (MRI) without enhancement revealed an 85 mm left ovarian tumor with irregular-shaped septum. T1-weighted images demonstrated slightly high signal intensity corresponding to the capsule of the cyst, as well as shading within the cyst, suggesting hemorrhagic contents (Figure 1(a)). Heterogeneous signal intensity at the posterior wall of the cyst, indistinguishable from solid tissue or clotting because of nonenhancement, was recognized on T2-weighted images (Figures 1(b) and 1(c)). Diffusion-weighted images demonstrated slightly high signal intensity of the whole tumor, but low signal intensity of the septum (Figure 1(d)). These findings suggested the possibility of malignancy. However, FDG-PET demonstrated a maximum standardized uptake value of 2.9 in the cyst (Figures 2(a) and 2(b)), which suggested a benign tumor rather than malignancy.

**Figure 3:**
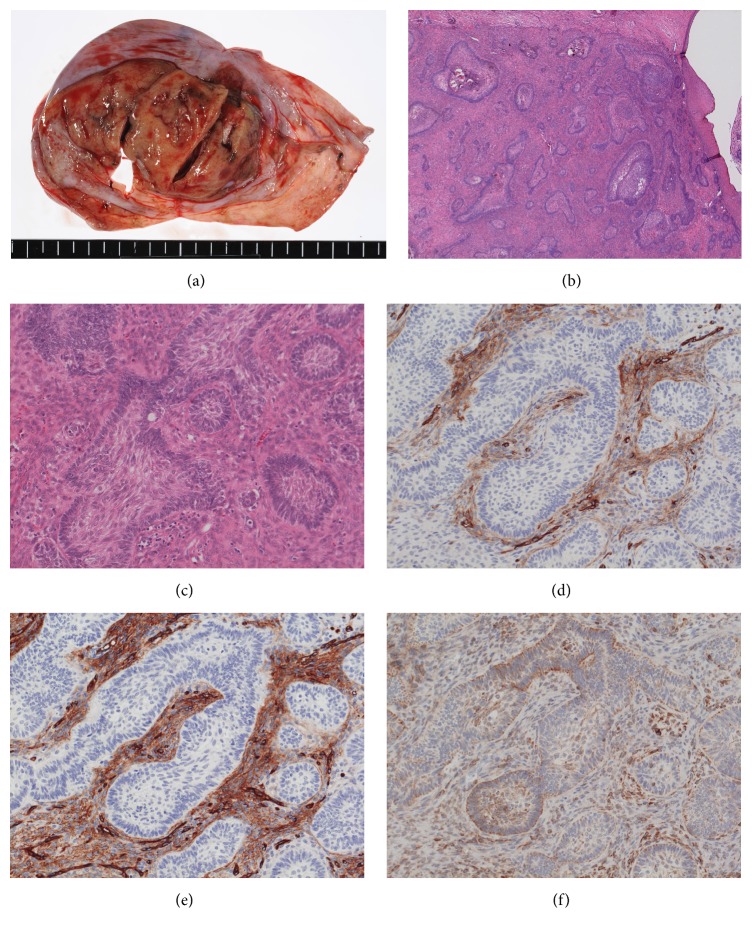
Macroscopic and pathologic findings. (a) Macroscopic findings. (b) Hematoxylin and eosin staining (magnification ×30). (c) Hematoxylin and eosin staining (magnification ×150). (d) Immunohistochemistry, showing positive results for CD10 (magnification ×150). (e) Immunohistochemistry, showing positive results for CD34 (magnification ×150). (f) Immunohistochemistry, showing positive results for Bcl-2 (magnification ×150).

We performed laparotomy with left salpingooophorectomy. Macroscopically, the left ovary had a unilocular cystic lesion, which contained a solid portion, approximately 4 cm in diameter, inside the cyst wall (Figure 3(a)). Histologically, the cystic lesion was lined by a stratified squamous epithelium and ciliated columnar cells and contained smooth muscle fibers and adipose tissue inside the cyst wall, which are compatible findings of MCT. The solid portion beneath the epithelium demonstrated well-circumscribed epithelial and mesenchymal proliferation (Figure 3(b)). The epithelial component showed lobular epithelial nests of basaloid cells, which were similar to hair germ. The epithelial nests were marginated by a peripheral nuclear palisade. The epithelial tumor cells exhibited neither high mitotic activity nor severe atypia. Fibroblastic cells proliferated in the stroma. There was no cleft formation between the epithelial nests and stroma (Figure 3(c)). Immunohistochemically, CD10 (Figure 3(d)) and CD34 (Figure 3(e)) were expressed in the stroma around the epithelial nests, and Bcl-2 was expressed in both the stroma and epithelial nests (Figure 3(f)). Based on these findings, we reached a diagnosis of TE arising in an ovarian MCT.

After surgery, she did not receive any additional treatment, such as chemotherapy or radiotherapy. After five years of follow-up, she is doing well and has had no sign of recurrence.

## 3. Discussion

We reported an extremely rare case of TE arising in an ovarian MCT, which was confirmed by pathologic findings with supplementary FDG-PET findings. Such examinations may be helpful for differentiating TE from BCC, especially in case of difficulty in conducting a biopsy.

TE is a benign epithelial tumor originating from the outer root sheath of a hair follicle, which has definitive follicular differentiation and predilection sites including the nose, upper lip, and cheeks. On the other hand, BCC is an epithelial tumor arising from progenitor cells of the interfollicular epidermis and upper infundibulum [1]. Clinical differentiation of TE from BCC can be difficult in some cases [2–7]. However, distinction between the two neoplasms is important because they have different biologic behavior and require different treatments.

TE and BCC are composed of nests of basaloid cells with follicular differentiation. Histologic differentiation of BCC from TE has been predominantly based on degree of follicular differentiation. A high degree of follicular differentiation favors a benign tumor, such as TE. Other typical histologic characteristics of TE include presence of primitive epithelial structures resembling hair papillae, known as papillary-mesenchymal bodies, and presence of small keratinous cysts. Conversely, BCC is characterized by cleft formation between the tumor and stroma, peripheral palisading of basaloid keratinocytes, inflammatory response, mitotic figures, necrosis, peritumoral mucin production, and occasional ulceration in the overlying squamous epithelium [5].

In our case, the lesion was well circumscribed and composed of epithelial nests of basaloid cells, without definite cleft formation between the epithelial nests and stroma or other histologic characteristics of BCC. However, pathologic examination can be confusing because TE and BCC have similar histologic characteristics. In this situation, immunohistochemistry is available for differential diagnosis of these tumors. In our case, the peritumoral stromal cells were immunoreactive for CD10, CD34, and Bcl-2, while the tumor cells were negative for CD10 and CD34 and diffusely positive for Bcl-2. These results support diagnosis of TE [6] because the peritumoral stromal cells in BCC are often negative for CD10 and CD34. The above-mentioned histopathologic criteria remain the best guideline for differential diagnosis of TE and BCC. However, Tebcherani et al. [6] reported that these two tumors could not be definitely differentiated by immunohistochemical examination alone.

The majority of malignant tumors arising in MCTs are squamous cell carcinoma or adenocarcinoma [8]. MRI, measurement of tumor markers (such as SCC and CA19-9) [15], and FDG-PET [16] may be useful for differentiating benign MCT from malignant transformation.

Our case is very different from previously reported cases of TE in terms of its histogenesis, that is, TE arising in an ovarian MCT. We had the opportunity to conduct an FDG-PET examination preoperatively to make differential diagnosis of ovarian tumor. Usually, in cases of typical dermatologic TE, FDG-PET is not conducted because of its benignity, and diagnosis is confirmed by biopsy. There are no previous reports regarding the FDG-PET findings of TE. However, there are numerous reports regarding the FDG-PET findings of BCC. One study showed that primary BCC lesions were not detected in three of six patients [10]. In the literature, they suggested that the histologic subtype of the BCC appeared to affect the ability of FDG-PET detection. Another review of 22 patients with metastatic BCC showed a mean maximum standardized uptake value of 7.3 (range, 1.9–16.8) [11]. There are only a few publications regarding imaging diagnosis of BCC. Komura et al. [12] reported the FDG-PET/CT findings in a case of prostatic BCC with lymph node and bone metastases. Both the primary tumor and metastases showed intense FDG uptake. Although there are no reports regarding the FDG-PET findings of TE arising in an ovarian MCT, considering that BCC is a malignant tumor and TE is a benign tumor, TE might demonstrate a weak standardized uptake value, as shown in our case.

In conclusion, we reported a rare case of TE arising in an ovarian MCT with immunohistochemical and FDG-PET findings. In case of difficulty in conducting the biopsy, like our case, FDG-PET may have informative findings before treatment, especially if the tumor showed positive FDG-PET findings; we can suspect that the tumor is malignant and we can prepare for the radicality of the surgery.
